# Regulation of Sleep by Neuropeptide Y-Like System in *Drosophila melanogaster*

**DOI:** 10.1371/journal.pone.0074237

**Published:** 2013-09-11

**Authors:** Chunxia He, Yunyan Yang, Mingming Zhang, Jeffrey L. Price, Zhangwu Zhao

**Affiliations:** 1 College of Agriculture and Biotechnology, China Agricultural University, Beijing, P. R. China; 2 University of Missouri-Kansas City, Kansas City, Missouri, United States of America; Imperial College London, United Kingdom

## Abstract

Sleep is important for maintenance of normal physiology in animals. In mammals, neuropeptide Y (NPY), a homolog of *Drosophila* neuropeptide F (NPF), is involved in sleep regulation, with different effects in human and rat. However, the function of NPF on sleep in *Drosophila melanogaster* has not yet been described. In this study, we investigated the effects of NPF and its receptor-neuropeptide F receptor (NPFR1) on *Drosophila* sleep. Male flies over-expressing NPF or NPFR1 exhibited increased sleep during the nighttime. Further analysis demonstrated that sleep episode duration during nighttime was greatly increased and sleep latency was significantly reduced, indicating that NPF and NPFR1 promote sleep quality, and their action on sleep is not because of an impact of the NPF signal system on development. Moreover, the homeostatic regulation of flies after sleep deprivation was disrupted by altered NPF signaling, since sleep deprivation decreased transcription of NPF in control flies, and there were less sleep loss during sleep deprivation and less sleep gain after sleep deprivation in flies overexpressing NPF and NPFR1 than in control flies, suggesting that NPF system auto-regulation plays an important role in sleep homeostasis. However, these effects did not occur in females, suggesting a sex-dependent regulatory function in sleep for NPF and NPFR1. NPF in D1 brain neurons showed male-specific expression, providing the cellular locus for male-specific regulation of sleep by NPF and NPFR1. This study brings a new understanding into sleep studies of a sexually dimorphic regulatory mode in female and male flies.

## Introduction

Sleep, consisting of a period of sustained quiescence that is associated with an increased arousal threshold, is a common phenomenon that widely exists in animals from vertebrates to invertebrates [1]. Flies share with mammals a similar sleep regulatory mechanism that involves two relatively independent processes: the circadian system that is responsible for consolidating sleep during the night and the homeostatic system that is responsible for wakefulness and sleep drive. Longer waking periods lead to longer and more intense sleep periods [2]. Sleep regulation in *Drosophila melanogaster* is anatomically located in the mushroom body, known to be involved in learning and memory [3], while approximately 150 clock neurons in the central nervous system are involved in setting circadian rhythms. These clock neurons are divided into three lateral neuron groups - dorsolateral neurons (LN _d_s), PDF-positive ventrolateral neurons (LN _V_s) and the fifth small ventrolateral neuron (5^th^ small LN_v_) - and three dorsal neuron groups - dorsal neurons 1, 2, and 3 (DN1, DN2, DN3). The LN _v_s in the circadian neuronal system contribute to sleep regulation by promoting wakefulness controlled by the GABA receptor and PER protein [4,5,6]. Dorsal neurons function in modulating sleep suppression during starvation [7].

Neuropeptide Y (NPY), a 36-amino-acid peptide from the pancreatic polypeptide (PP) family, is one of the more abundant peptides in the central and peripheral nervous system in mammals [8]. NPY is involved in several physiological functions, such as food intake, hormonal release, circadian rhythms, cardiovascular disease, thermoregulation, stress response and anxiety [9,10,11,12]. In humans, NPY enhances sleep by prolonging the sleep period and reducing sleep latency and wakefulness [13]. In contrast, NPY in rats increases wakefulness and decreases sleep [14]. These findings indicate that NPY regulates sleep differently in different animal species, probably due to their diurnal or nocturnal difference. NPY acts through its trans-membrane receptor (a G-protein-coupled receptor) and through Gi/o signaling pathways (mediated by α or βγ subunits) to inhibit cAMP formation and intracellular Ca^2+^ mobilization, and to modulate Ca^2+^ and K^+^ channels [15].

In invertebrates, neuropeptide F (NPF) and its receptor (NPFR1 is the only identified functional receptor for NPF in *D. melanogaster*) show conserved structure and function with NPY and NPY receptors in mammals [16]. NPF in *D. melanogaster* has been characterized for involvement in diverse behavioral responses, such as food intake, hypermobility, cooperative burrowing, alcohol sensitivity and locomotor rhythm [17,18,19,20]. However, regulation of sleep by NPF in invertebrates has not yet been characterized. In this study, we explored NPF function in sleep by over-expressing *npf* and *npfr1* in brain-specific neurons.

## Materials and Methods

### Fly strains

The *npf-gal4*, *npfr1-gal4*, UAS-*npf*, UAS-*npf* ^*dsRNA*^, UAS-*npfr1*, UAS-*npfr1* ^*dsRNA*^, and UAS-mCD8-GFP strains have been previously described [18,21,22]. The tubP-GAL80[ts] (stock NO. 7018) was purchased from the Bloomington Drosophila Stock Center (Indiana University). The clk 8.0-Gal4 flies were from the laboratory of Dr. Paul E. Hardin (Texas A&M University, Texas, U.S.). Flies were reared on a standard cornmeal–yeast–agar medium at 25°C and 65% relative humidity in a 12 hr L:12 hr D cycle.

### Sleep analysis and statistics

Flies were placed in 65 mm × 5 mm glass tubes containing standard fly food at one end. They were acclimated in behavior tubes for at least 24 hr at 25°C in LD conditions, and then data were collected in LD for 4 days with the DAM System (Trikinetics, Waltham, MA) in 1-min bins. Sleep parameters were measured with Pysolo software (obtained from the website: http://www.pysolo.net) using averages over 4 days of LD [23]. Sleep deprivation was performed using a mechanical method to keep flies awake for 12 hours during the night after 3 days of baseline measurement [24]. Statistical analysis was performed in a SPSS program (SPSS, Chicago, IL, USA). Significance levels were determined by independent *t*-test, and ^*^ indicates *p* < 0.05, ^**^ indicates *p* < 0.01, and ^***^indicates *p* < 0.001.

Mean sleep episode duration and sleep bout number are important for analysis of sleep quality [25]. Sleep latency - the time for flies to fall asleep once the lights are turned off or on during LD cycles - reflects the sleep pressure, which influences sleep-initiation neuronal firing [26]. Here, sleep latency was measured and analyzed from the time of lights off or lights on to the onset of the first sleep episode as previously reported [26].

### Immunohistochemistry

NPF-expressing cells were identified through immunofluorescence methods in female and male brains as described previously [20]. The brain samples were viewed with Nikon ECLIPSE TE2000-E and Nikon D-ECLIPSE (Nikon, Tokyo, Japan) confocal microscopes. Confocal images were obtained at an optical section thickness of 1–2 µm, and NPF immunofluorescence was quantified by ImageJ (http://rsb.info.nih.gov/ij/index.html) as previously described [20].

### Quantitative real-time PCR for measurements of npf expression level

*Npf* expression level was analyzed as described in a previous report [20]. *Npf* amount was determined by the mean of three independent replicates. Significant differences were determined as mentioned above.

## Results

### The efficiency of Gal4-UAS transgenic system for npf and npfr1 expression

The *npf-gal4, UAS-npf, npfr1-gal4*, *UAS-npfr1, UAS-npf* ^*dsRNA*^ *i and UAS-NPFR* ^*dsRNAi*^ lines have been widely used for regulation of *npf* and *npfr1* expression [18,27]. Our experiments further demonstrated a one and a half - two-fold increase at the transcript level in *npf* and *npfr1* over-expression lines, and a 50% decrease in *npf* and *npfr1* knockdown (dsRNAs) lines (Figure S1).

### Sleep regulation by NPF and NPFR1 in *D. melanogaster*

**Figure 1:**
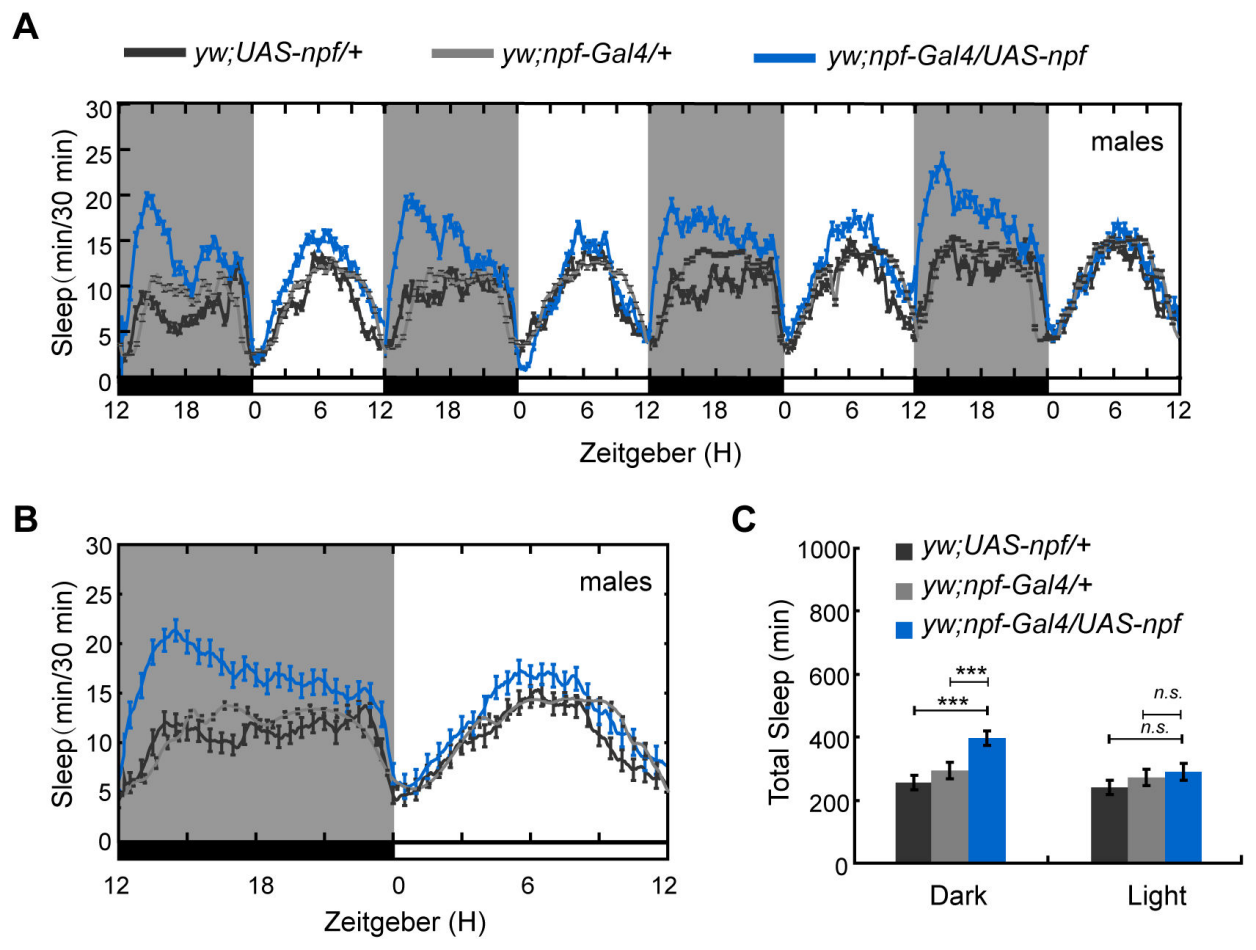
Sleep in male flies with up-regulated *npf*. Sleep during the nighttime was significantly increased when *npf* expression was enhanced (n=60 for controls, and 62 for *yw; npf-Gal4/UAS-npf*). (A) Daily sleep profile that showed increased sleep during the nighttime in *npf* over-expressing line. (B) Average daily sleep profile over 4 days that revealed increased sleep during the night. (C) Statistical analysis of sleep. Black and gray histograms indicate the controls, and blue histograms indicate the up-regulated *npf* line. Black and gray lines indicate controls, and the blue line represents the up-regulated *npf* line. Error bars indicate the SEM. White bars indicate lights on; black bars indicate lights off. ***, p<0.001; *, p<0.05; *n.s.*, no significant differences.

**Figure 2:**
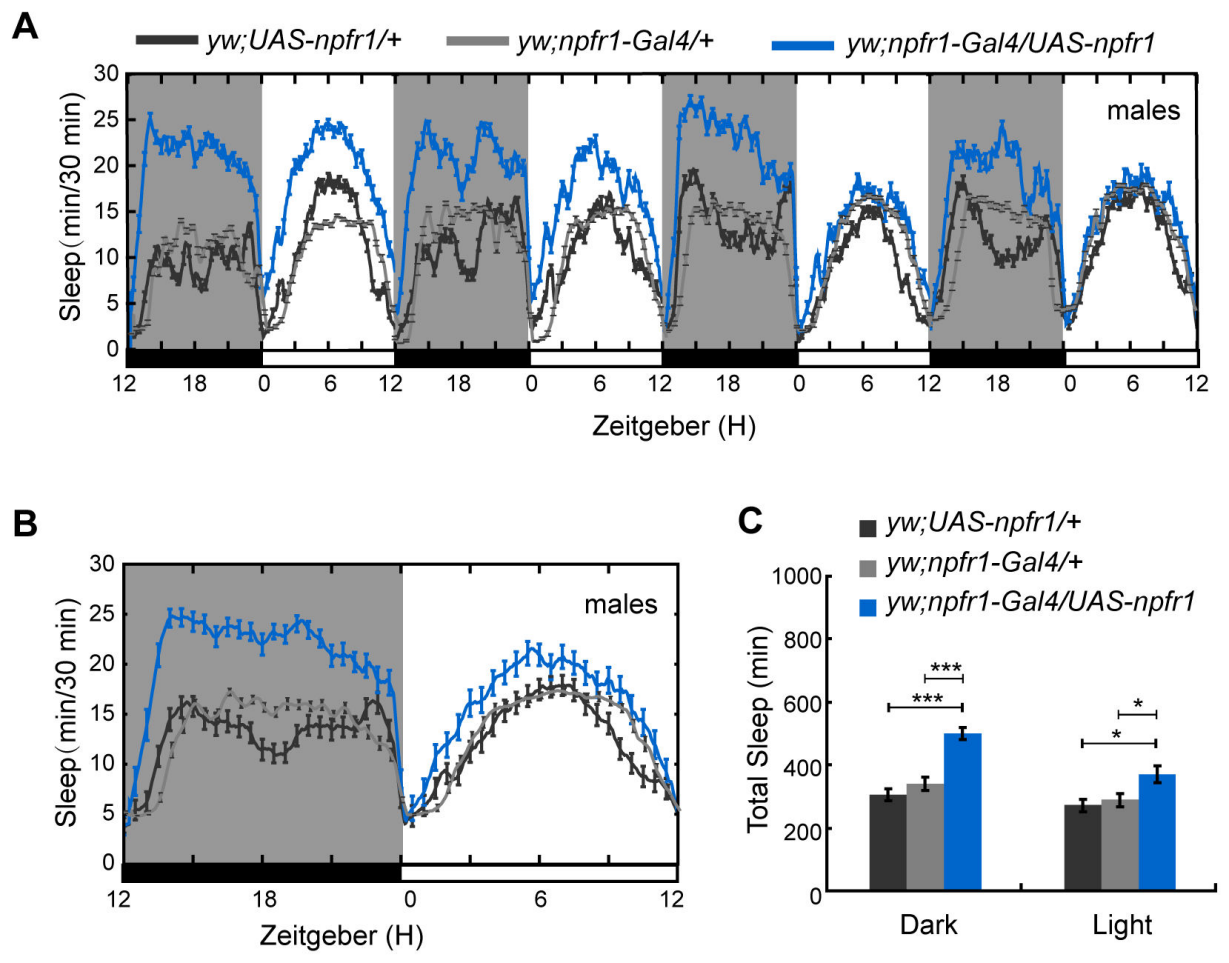
Sleep in male flies with up-regulated *npfr1*. Sleep during the nighttime was significantly increased when *npfr1* expression was enhanced (n=61 for controls, and 51 for *yw; npfr1-Gal4/UAS-npfr1*). (A) Daily sleep profile that showed increased sleep during the nighttime and the daytime in an *npfr1* over-expressing line. (B) Average daily sleep profile over 4 days that revealed increased sleep during the night and the day. (C) Statistical analysis of sleep. Labeling is the same as that described in Figure 1.

**Figure 3:**
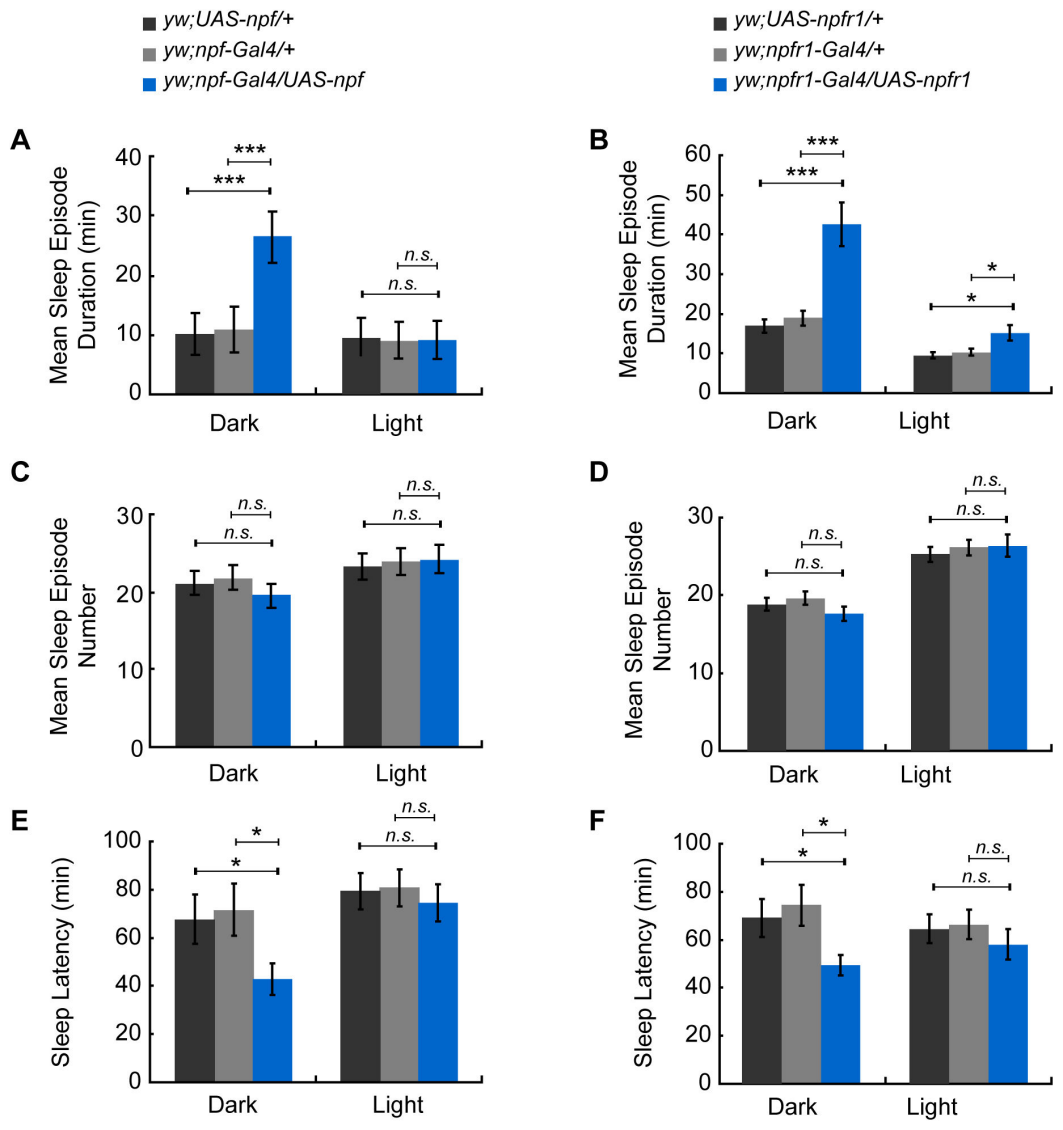
Analysis of sleep quality and quantity in male flies with up-regulated *npf* and *npfr1*. (A–B) Analysis of sleep episode duration for *npf* and *npfr1*. (C–D) Analysis of sleep episode number for *npf* or *npfr1*. (E–F) Analysis of sleep latency for *npf* and *npfr1*. Labeling is the same as that described in Figure 1.

To investigate the role of NPF and NPFR1 in sleep regulation of *D. melanogaster*, we first used the Gal4-UAS system to increase or decrease *npf* and *npfr1* expression in NPF- and NPFR1-expressing cells in the brain, and detected the sleep amount both in females and males. Under 12 hr L: 12 hr D conditions (LD), male flies with *npf* over-expression slept more, with a significant increase in whole nighttime sleep (mean±SEM: 392±22 min for *npf* over-expression line and 221±18 and 292±23 min for UAS and Gal4 controls) (*p* < 0.001) but not daytime sleep (Figure 1), and males with over-expressed NPFR1 also increased sleep both during daytime (mean±SEM: 350±24 min for *npfr1* over-expression line and 241±11 and 243±13 min for UAS and Gal4 control lines) (*p* < 0.05) and nighttime (mean±SEM: 506±19 min for *npfr1* over-expression line and 311±15 and 333±16 min for UAS and Gal4 control lines) (*p* < 0.001) (Figure 2). Furthermore, the sleep increase at nighttime was due to a significant increase in sleep episode duration in the males of overexpressing *npf* (mean±SEM: 26.56±4.16 min for *npf* over-expression line and 10.03±3.21 and 10.89±3.34 min for UAS and Gal4 control lines) (*p* < 0.001) but not in sleep bout number (Figure 3A, C) and the males of overexpressing *npfr1* (mean±SEM: 42.68±5.49 min for *npfr1* over-expression line and 17.33±1.79 and 19.86±2.01 min for UAS and Gal4 control lines) (*p* < 0.001) (Figure 3B & D). Further analysis showed that the sleep latency after lights off was significantly decreased in both the *npf*- and *npfr1*- over-expression lines, indicating a higher pressure to fall asleep (Figure 3E-F) (mean±SEM: 42.75±6.51 and 49.45±4.38 min for *npf* and *npfr1* over-expression lines, 67.69±10.13 and 70.38±10.75 min for *npf* over-expression UAS and Gal4 control lines, and 68.92±7.75 and 74.83±7.26 min for *npfr1* over-expression UAS and Gal4 control lines) (*p* < 0.05). However, sleep in males with *npf* and *npfr1* down-regulated expression (Figure S2) and in females with both up- and down- regulated expression (Figure S3) was not affected in comparison with sleep in the controls. These data indicate that the NPF neuronal system regulates sleep in a sex-dependent manner.

**Figure 4:**
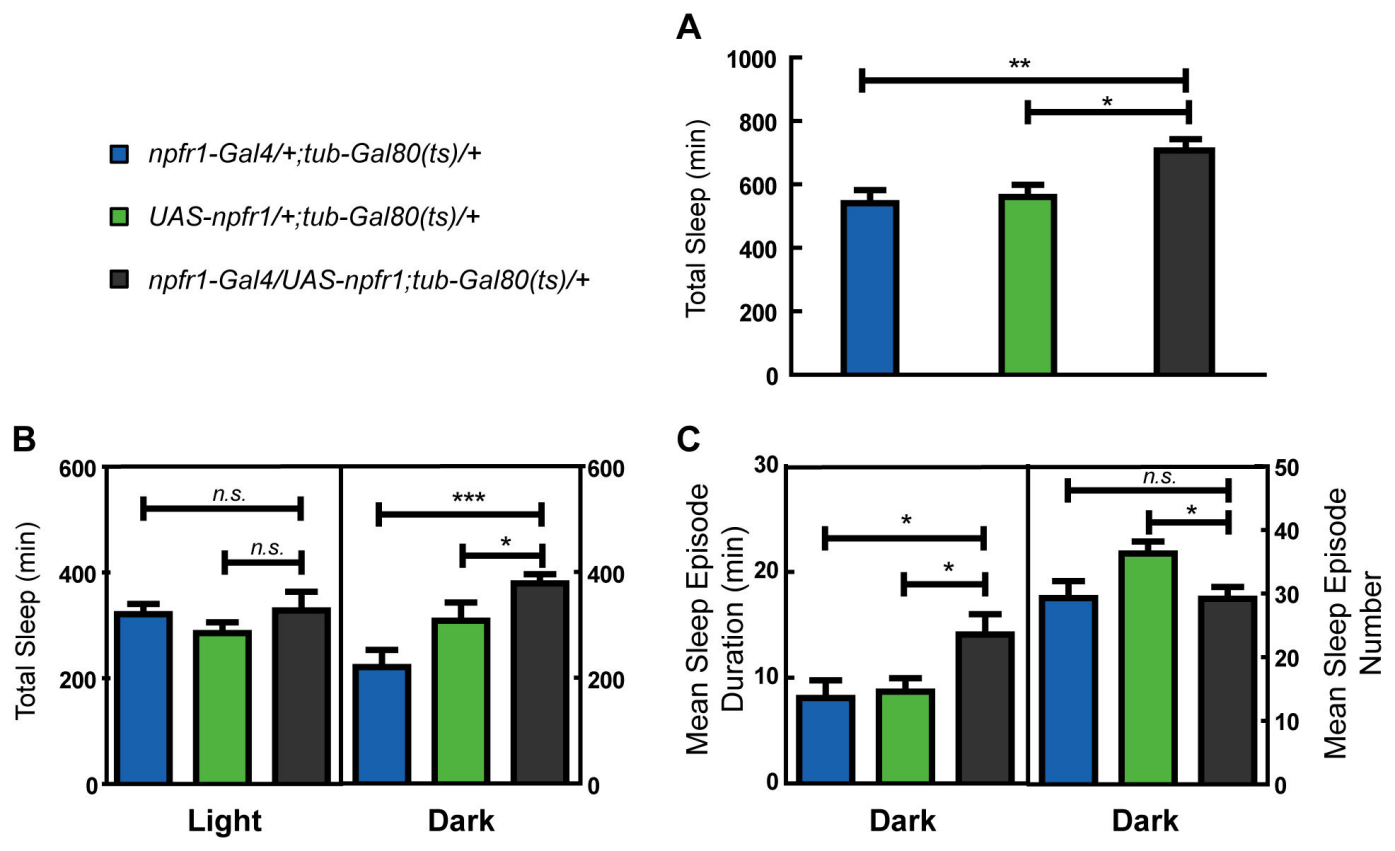
Analysis of sleep in male flies with up-regulated *npfr1* during adulthood. Larvae and newly emerged adults were reared at 18°C, and newly emerged flies were entrained for three LD cycles at 18^o^C and then transferred to 30°C to block Gal80, in order to activate Gal4-driven gene expression. (A) Total sleep was increased in *npfr1* over-expression flies (n=15 for both the controls and n=13 for *npfr1-Gal4/UAS-npfr1;tub-Gal80*(ts)). (B) Daily sleep profile showed increased sleep during nighttime in an *npfr1* over-expressing line. (C) Sleep increase during nighttime was mainly derived from increased sleep episode duration. Error bars and significant difference are as described above.

To exclude any impact of increased NPF signaling on embryonic and larval development, *npfr1* was over-expressed only in adults. Larvae and newly emerged adults were reared at 18°C to express an active Gal80 protein that represses the activity of Gal4. Newly emerged flies were entrained for three LD cycles at 18^o^C and then transferred to 30°C to block Gal80, in order to activate Gal4-driven gene expression. Results showed that over-expression of *npfr1* only in adults can increase total sleep time, caused by a significant increase in sleep during the night (Figure 4A & B). Further analysis showed that the increase in nighttime sleep was mainly derived from an increased sleep episode duration (Figure 4C), consistent with the results above.

### Influence of NPF and NPFR1 on sleep homeostasis

**Figure 5:**
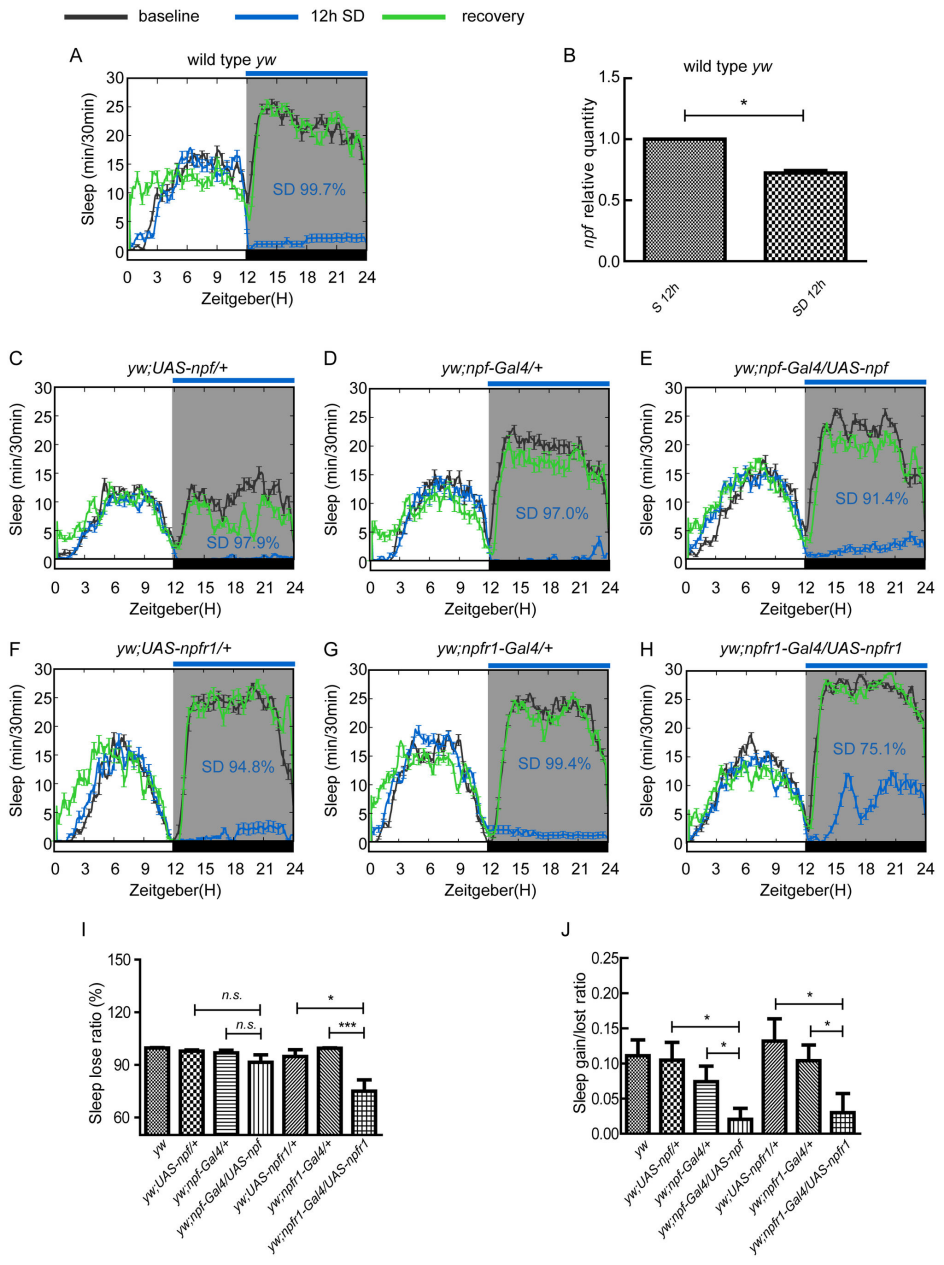
The homeostatic sleep response regulated by NPF signal in males. (A) Sleep rebound after 12 hour sleep deprivation in wild type flies (yw) is depicted. (B) *npf* expression was reduced after 12 hour sleep deprivation. (C, D and E) NPF regulated sleep homeostasis, in which C and D are controls for E. Sleep rebound was reduced when *npf* expression was enhanced (n=19-22 for controls, and n=20 for *yw; npf-Gal4/UAS-npf*). (F, G and H) NPFR1 regulated sleep homeostasis, in which F and G are controls for H. Sleep rebound was reduced when *npfr1* expression was enhanced (n=21-24 for controls, and n=23 for *yw; npfr1-Gal4/UAS-npfr1*). Error bars and significant differences are as described above. (I) Sleep loss is reduced after sleep deprivation in *npf* and *npfr1* up-regulated lines. (J) Sleep gain/lost ratio is reduced after sleep deprivation in *npf* and *npfr1* up-regulated lines. Error bars and significant difference are as described above.

Homeostatic regulation is a key feature of sleep [28]. To investigate whether NPF and NPFR1 have a function in sleep homeostasis, we employed sleep deprivation experiments using a mechanical sleep deprivation method as previously mentioned [24]. One of the key features for sleep homeostatic regulation is that an obvious sleep rebound occurs after sleep deprivation (Figure 5A). We measured the NPF transcript in flies both with and without sleep deprivation, and found that *npf* expression in the heads of adult flies was down-regulated after sleep deprivation for 12h (Figure 5B), suggesting that NPF signaling plays an important role in sleep homeostasis. Consistent with such a role, when *npf* expression was up-regulated, flies exhibited less sleep loss (Figure 5I: 91.4% in an *npf* up-regulated line compared to 97.9% and 97.0% in the UAS and Gal4 control lines), and sleep loss was significantly reduced in *npfr1*up-regulated lines (Figure 5I: 75.1% in *npfr1* up-regulated line compared to 94.8% in UAS control line (*p* = 0.03) and 99.4% in Gal4 control line (*p* = 0.0003)). Correspondingly flies with up-regulated *npf* and *npfr1* expression showed smaller sleep rebound after sleep deprivation than did the controls (Figure 5 C–H). The sleep rebound for *npf* up-regulated flies was 13.24±4.93, which was smaller than UAS (25.18±5.81, *p* = 0.13) and Gal4 controls (29.00±9.31, *p* = 0.12) although the differences were not statistically significant. The sleep rebound for *npfr1* up-regulated flies was 15.63±6.93, which was significantly smaller than UAS (57.00±10.831, *p* = 0.024) and Gal4 controls (32.90±7.31, *p* = 0.038). The sleep gain/loss ratio in flies with up-regulated *npf* and *npfr1* expression was also lower (0.02±0.01 for the *npf* up-regulation line, and 0.03±0.02 for the *npfr* up-regulation line) than the controls (0.10±0.03, 0.07±0.02 for *npf* up-regulation UAS and Gal4 controls, and 0.13±0.03, 0.10±0.02 for *npfr1* up-regulation UAS and Gal4 controls) (Figure 5J). These results indicate that NPF and NPFR1 play an important role in the homeostatic regulation of sleep.

### Npf levels in male and female flies during nighttime

**Figure 6:**
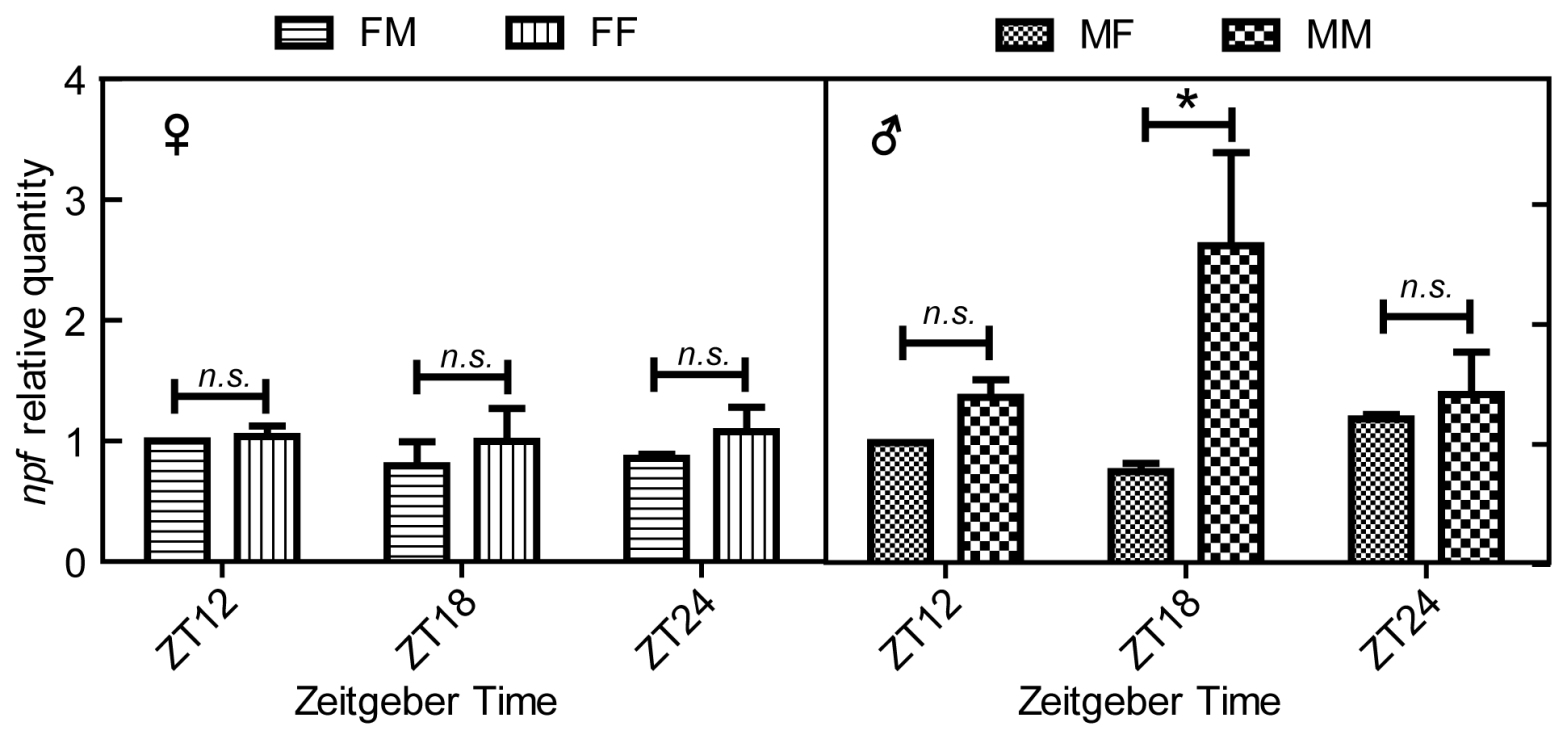
Brain *npf* levels in male and female flies. (A) *npf* levels in female flies with the presence of males (FM) and female flies without the presence of males (FF): (B) *npf* levels in male flies with the presence of females (MF) and male flies without the presence of females (MM). Error bars and significant differences are as described above.

Previous studies showed that the nocturnal phase of *Drosophila* courtship behavior is dependent on the male and not the female circadian clock [29], indicating there exists a sex-specific regulation pathway to balance sleep and mating behavior in male flies. Might NPF signaling in males participate in this pathway? Is NPF signaling decreased to suppress sleep for mating behavior in males when females are present or increased to promote sleep in males when females are not present? To explore these questions, we analyzed the *npf* expression levels in wild type males and females flies during nighttime with the presence of opposite sex or same sex couples. Results showed that the *npf* expression levels in female flies were not changed whether male flies were present or not (Figure 6). In contrast, when females were present, male flies expressed a lower *npf* level at midnight (ZT18) that could promote increased activity for courtship behavior, and when females were not present males expressed a higher level of *npf* that could promote sleep (Figure 6).

### The neuronal localization of NPF

**Figure 7:**
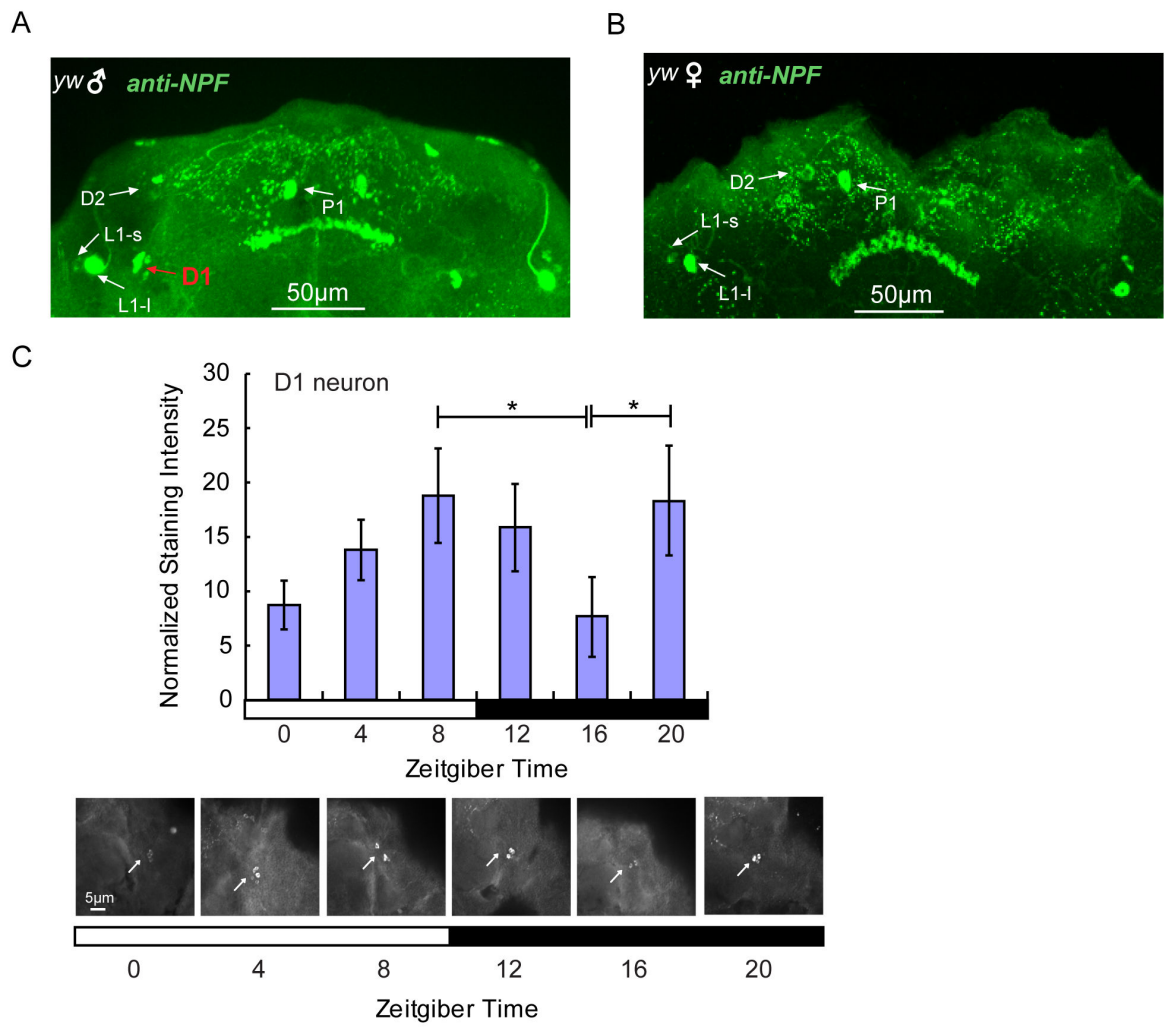
Male specific expression of NPF in male brain. (A) anti-NPF labeled NPF localization in male brains in which D1 is male-specific. (B) NPF localization in female brains in which D1 is not detected. (C) NPF oscillation in male-specific D1 neurons. The arrows in the images represent the staining intensity of NPF at ZT0, ZT 4, ZT8, ZT12, ZT16 and ZT20. The ZT8 and ZT20 are compared with ZT16 for statistical difference analysis (*, *p* < 0.05). Scale bar: 5 µm for neuron figures.

We found in this study that sleep regulation by NPF in flies is sex-dependent. What are the structures that mediate sex-dependent regulation of sleep? Previous reports showed that NPF was expressed in two groups of dorsal neurons (D1 and D2), two groups of dorsomedial neurons (P1 and P2), two groups of dorsolateral neurons (L1-l and L1-s), one lateroventral group (L2), one subesophageal group (L2 and S) and the fan-shaped body (FB) [20,27,30]. Interestingly, NPF expression was male-specific in 2-4 D1 noncircadian neurons (Figure 7A & B). Furthermore, daily expression of NPF in D1 neurons exhibited two daily peaks - one at ZT8 and another at ZT20 (Figure 7C). The peak at ZT20 is consistent with the *npf* mRNA peak at ZT18 (Figure 6) in males like these in Figure 7C, which are isolated from females at this time.

## Discussion

In this study, we analyzed the function of NPF and NPFR1 on the sleep phenotype in *Drosophila* and found that NPF and NPFR1 both promote sleep during the night in male flies, in a similar fashion to NPY’s effect in humans. Either NPF or NPFR1 over-expression causes an increase in nighttime sleep by increasing average sleep episode duration and reducing sleep latency during the night. NPF and NPFR1 also play an important role in the homeostatic regulation of sleep. NPF and NPFR1 may regulate sleep in *Drosophila* because NPFR1 activated by NPF inhibits cAMP formation [15]. The activity of cAMP-dependent PKA has been reported to be involved in sleep regulation in *Drosophila* [3,31].

Sexually dimorphic behaviors are widely observed in invertebrate and vertebrate species. Locomotor activity in *Drosophila* was found to show sexual dimorphism [32,33]. However, sexually dimorphic regulation of sleep has rarely been reported. Sleep in the *cyc* ^*01*^ mutant fly is sexually dimorphic with reduced or absent locomotion in males and exaggerated locomotion in compensatory rebound after sleep deprivation in females [34]. Fujii et al. have found a male-specific function in courtship, indicating a male-specific signal circuit might function in this sexually dimorphic behavior [30]. The NPF signal system is shown to participate in the circuit in this paper. In *Drosophila* , sexual differentiation is ultimately dependent on a chromosomal signal that is different between males (XY) and females (XX), resulting in repression or activation of the key gene *Sexlethal* (*Sxl*), responsible for dosage compensation, somatic sexual development, oogenesis and sexually dimorphic neural development. The subordinate gene *transformer (tra)* is one of the downstream regulatory genes of *Sxl* that controls sex determination [35]. The expression of *npf* in *Drosophila* is regulated in both sex-nonspecific and male-specific (ms) manners, and the *ms-npf* expression is controlled by the *tra*-dependent sex-determination pathway [27], indicating NPF is regulated by the sexual differentiation pathway. The male-specific NPF brain neurons (D1) may provide the neuronal locus for male-specific regulation of sleep.

The circadian system is involved in fly sleep regulation, and the circadian pacemaker neurons participate in regulating sleep. Some NPF in fly brains is found in clock neurons including the LN _v_s, LN _d_s and DN1s, among which the LN _v_s have been shown to promote wakefulness [4,5,6], while the dorsal circadian neurons modulate sleep suppression during starvation [7]. We found that NPF and NPFR1 promote sleep quality especially during the night by prolonging average sleep episode duration and reducing night sleep latency in males. While these effects are associated with D1-specific expression in males, it is possible that the clock-neuron specific expression in males also contributes to these effects, which are absent in females because the neural circuit is incomplete (i.e., clock-neuron specific expression is present but D1 expression is absent.).

In humans, sleep is restorative and plays a crucial role in long-term memory storage and learning. Sleep disorders cause a series of health problems associated with cognitive deficiency, poor job performance and low productivity [36]. In *Drosophila* , aging and diet are associated with sleep change [37,38]. Starvation suppresses sleep and sleep deprivation is known to affect longevity [7,39]. Therefore, it is likely that the function of NPF involves regulation of interactions among feeding, sleep, copulation and even longevity.

## Supporting Information

Figure S1 **Detection of expression in the transgenic genotypes for *npf* and *npfr1* mRNAs by qRT-PCR.** The Gal4-driven expression of *npf* and *npfr1* increased transcripts by 1.5 to 2 fold. Double strand RNA (dsRNA) for *npf* and *npfr1* expression caused about a 50% decrease in expression.
(TIF)

Figure S2 **Sleep in male flies with down-regulated *npf* and *npfr1*.** (A) Average daily sleep profile over 4 days in male flies with down-regulated *npf* (n=31 for each control, and 32 for *yw; npf-Gal4/UAS-npf* ^*dsRNA*^). (B) Statistical analysis of sleep. (C) Average daily sleep profile over 4 days in male flies with down-regulated *npfr1* (n=32 for each control, and 36 for *yw; npf-Gal4/UAS-npf* ^*dsRNA*^). (D) Statistical analysis of sleep. Labeling is the same as that described in Figure 1.
(TIF)

Figure S3 **Sleep is unchanged when *npf* or *npfr1* expression is up- or down-regulated in female flies.** (A) Total sleep in *npf* over-expressing females is maintained at a similar level with sleep in the control flies except for a small increase before lights on (n=32 for controls, and 34 for *yw; npf-Gal4/UAS-npf*). (B) The total sleep in *npf*-down-regulated females is not changed either (n=32 for controls, and 36 for *yw; npf-Gal4/UAS-npf* ^*dsRNA*^). (C) Total sleep in females with *npfr1* over-expression is similar to that in control flies (n=54 for controls and 49 for *yw; npfr1-Gal4/UAS-npfr1*). (D) The total sleep in *npfr1*-down-regulated females is not changed (n=59 for controls, and 58 for *yw; npfr1-Gal4/UAS-npfr1* ^*dsRNA*^). Linear graphs, error bars, white and black bars, and white and gray background are as indicated in Figure 1.
(TIF)
