# Supplementary figures and images for: Regulation of Sleep by Neuropeptide Y-Like System in Drosophila melanogaster
Source: PLoS One. 2013 Sep 11;8(9):e74237. doi: 10.1371/journal.pone.0074237 (PMC3770577; doi:10.1371/journal.pone.0074237)

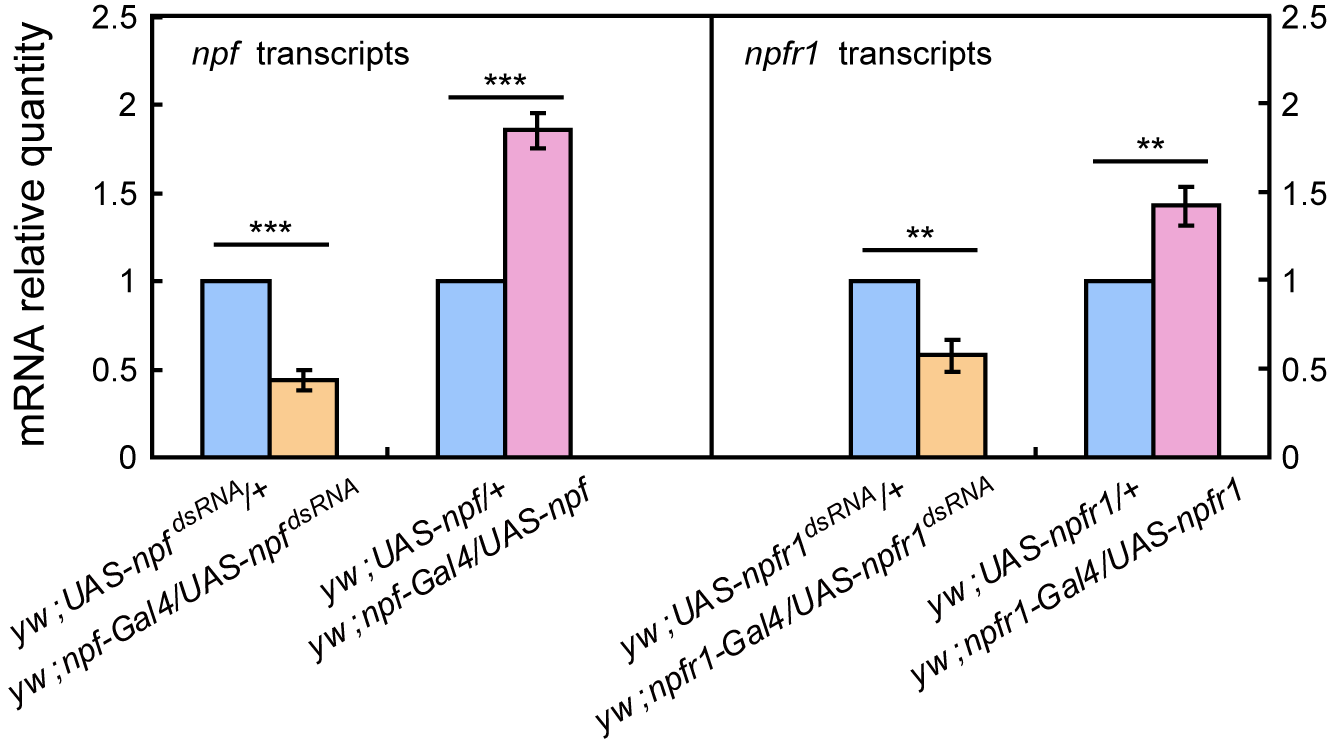

Supplement: Figure S1 — Detection of expression in the transgenic genotypes for npf and npfr1 mRNAs by qRT-PCR. The Gal4-driven expression of npf and npfr1 increased transcripts by 1.5 to 2 fold. Double strand RNA (dsRNA) for npf and npfr1 expression caused about a 50% decrease in expression. (TIF) [file pone.0074237.s001.tif]

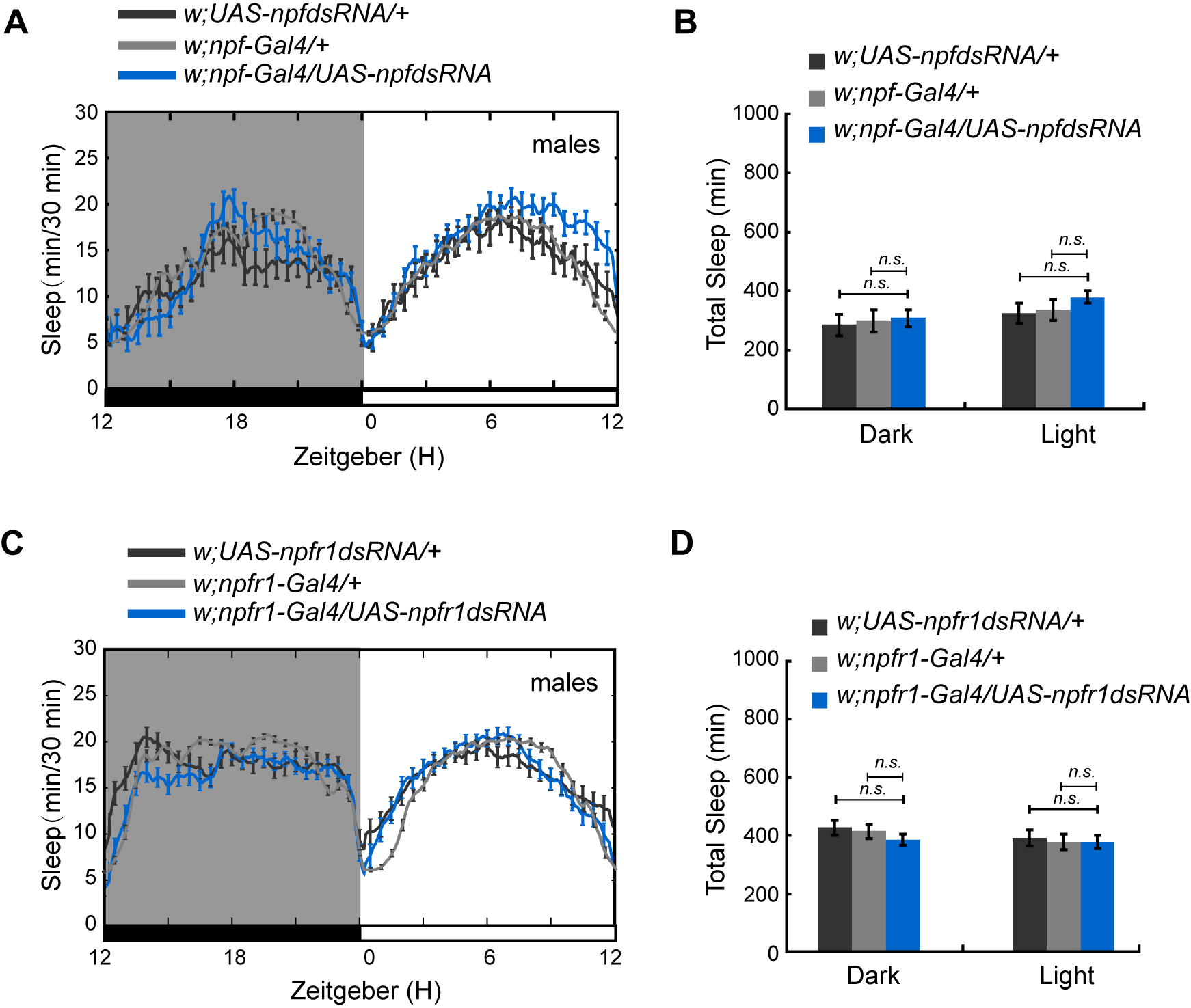

Supplement: Figure S2 — Sleep in male flies with down-regulated npf and npfr1. (A) Average daily sleep profile over 4 days in male flies with down-regulated npf (n=31 for each control, and 32 for yw; npf-Gal4/UAS-npf dsRNA). (B) Statistical analysis of sleep. (C) Average daily sleep profile over 4 days in male flies with down-regulated npfr1 (n=32 for each control, and 36 for yw; npf-Gal4/UAS-npf dsRNA). (D) Statistical analysis of sleep. Labeling is the same as that described in Figure 1. (TIF) [file pone.0074237.s002.tif]

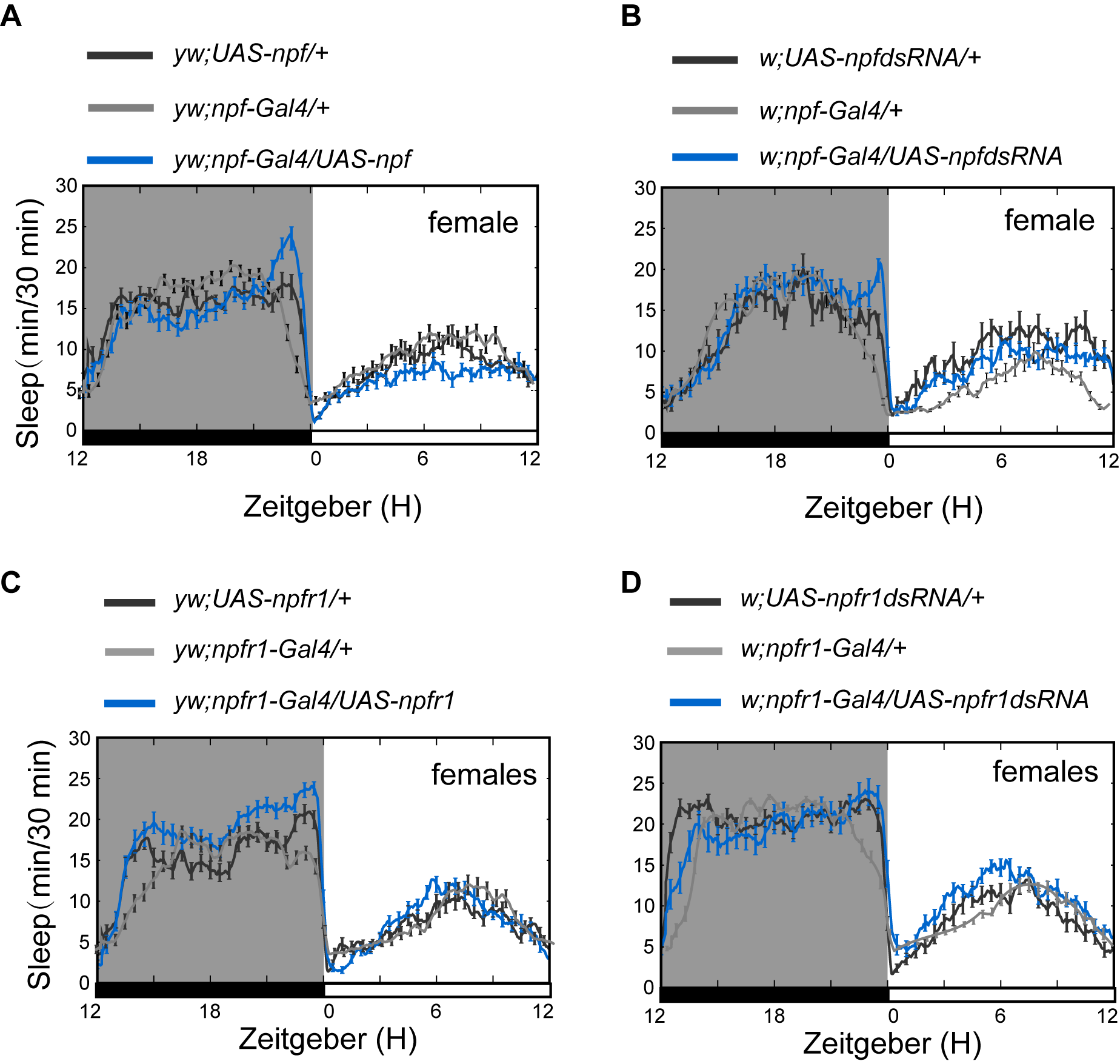

Supplement: Figure S3 — Sleep is unchanged when npf or npfr1 expression is up- or down-regulated in female flies. (A) Total sleep in npf over-expressing females is maintained at a similar level with sleep in the control flies except for a small increase before lights on (n=32 for controls, and 34 for yw; npf-Gal4/UAS-npf). (B) The total sleep in npf-down-regulated females is not changed either (n=32 for controls, and 36 for yw; npf-Gal4/UAS-npf dsRNA). (C) Total sleep in females with npfr1 over-expression is similar to that in control flies (n=54 for controls and 49 for yw; npfr1-Gal4/UAS-npfr1). (D) The total sleep in npfr1-down-regulated females is not changed (n=59 for controls, and 58 for yw; npfr1-Gal4/UAS-npfr1 dsRNA). Linear graphs, error bars, white and black bars, and white and gray background are as indicated in Figure 1. (TIF) [file pone.0074237.s003.tif]
